# Why consumers have impulsive purchase behavior in live streaming: the role of the streamer

**DOI:** 10.1186/s40359-024-01632-w

**Published:** 2024-03-06

**Authors:** Xiaolin LI, Dunhu Huang, Guofeng Dong, Bing Wang

**Affiliations:** 1 Business and Tourism School, Sichuan Agricultural University, 611830 Chengdu, China; 2 Informatics Research Centre, Henley Business School, University of Reading, RG6 6UD Reading, UK

**Keywords:** Live streaming, Streamer, Trust, Flow, Impulsive purchase behavior

## Abstract

To explore the influencing factors of consumers’ impulsive purchase behavior in live streaming, based on the Stimulus-Organism-Response framework, we construct the model of how streamers influence consumers’ impulsive purchase behavior by consulting literature. Collected data by means of a questionnaire, and made an empirical study by using the structural equation model to explore the mechanism of streamer affecting consumers’ impulsive purchase behavior. The results show that streamer characteristics (personal charisma, professionalism) and streamer performance (interactivity, entertainment) affect consumers’ impulsive purchase behavior by affecting consumers’ trust and flow experience. The empirical results have important theoretical and practical significance.

**Supplementary Information:**

The online version contains supplementary material available at 10.1186/s40359-024-01632-w.

## Introduction

As a hot spot in recent years, “live shopping” has gradually integrated into the lives of consumers, changed the traditional buying habits of the public, and become an effective marketing means for major platforms. Since 2017, China’s live streaming e-commerce market has grown rapidly, and the popularity of live commerce has continued to rise. With the continuous popularization of 5G network technology in China in recent years, live streaming technology has also brought innovation and promoted the upgrading of the live streaming industry. China has gone through the initial development stage of live streaming e-commerce. Twitch admitted that China’s live streaming sales market was at “another level”, and the growth rate is faster than other parts of the world [1]. The live streaming industry in china has entered a standardized stage, which is very representative in the global live streaming industry. However, to achieve the refined and stable development of live streaming e-commerce, we still need to pay attention to the details of the live streaming industry.

According to the results of 《the 47th China Statistical Report on Internet Development》 released by CNNIC in 2021, as of December 2020, the users of e-commerce live streaming accounted for 39.2% of the overall internet users, which provided a good foundation for the development of live streaming, and the number of live streaming users is still increasing, indicating that there is a good space for the development of e-commerce live streaming in the future. Both e-commerce platforms and various social business media have taken live shopping as an important development direction. In the top 10 of the “iiMedia Report” in 2020 China live shopping streamer turnover list, it is found that the sales of online live streaming represented by “Li Jiaqi Austin” are 21.861 billion, which highlights the great value of streamers in live shopping. In E-commerce, how to better play the role of streamers is an important problem to be considered.

As an important core of “live shopping”, the streamer has a profound impact on consumers’ live shopping decisions [2]. U&G theory holds that people will choose media according to their own needs and motives. In live shopping, consumers are often affected by their utilitarianism, pleasure, and social satisfaction [3]. Streamers often play the role of opinion leaders through live streaming media, using their own charm to attract consumers, with the interaction of improving customer experience, and meet the demand of consumer hedonic and utilitarian, enlarging consumers’ real feelings visually and audibly, to enhance the sense of immersion, create a better virtual social atmosphere, and enhance the social satisfaction of consumers [4]. Some scholars have divided the attributes of e-commerce streamers that have a positive impact on consumers’ online purchase decisions into four categories: charm, recommendation, display, and interaction [5]. However, for live shopping streamers, credibility, professionalism, and interactivity are important factors to arouse consumers’ purchase [6]. Due to the uncertainty of the online environment, the trust in online purchases will be affected by more factors than offline purchases. Therefore, how to improve consumers’ trust in the live shopping scene has become a problem that must be considered. In addition, consumers’ experience in the purchase process is also very important. The performance of the streamer in the live streaming process directly affects consumers’ final purchase.

Impulse purchase behavior is a common phenomenon in live streaming. In the online survey report on consumer satisfaction with live e-commerce shopping released by the China Consumer Association in 2020, consumers believe that “impulsive consumption is very serious” accounts for the highest proportion, reaching 44.1%, which shows that impulsive purchase behavior occurs frequently in live shopping. For the live streaming platform, consumers’ impulsive purchase behavior in the live streaming is a new development direction. Making full use of this psychological mechanism can effectively promote the development of the live streaming platform. In the context of e-commerce, online impulsive purchase behavior focuses on consumers who lack corresponding resistance due to the stimulation in the online platform and finally produce spontaneous purchase behavior [7]. In the past, most scholars focused on the research of online impulse purchases from the perspective of online purchase environmental factors, network technology, marketing activities, and product types, but there were relatively few studies on consumers’ online impulse purchase behavior from the streamer perspective.

Therefore, based on the above background and research, and based on the SOR model widely used in consumer behavior research, combined with impulsive purchase behavior and streamer-related research, this paper constructs a theoretical model of the influencing factors of streamer on consumer impulsive purchase behavior in the context of online shopping. The model takes the characteristics and performance of the streamer as stimulus variables, and forms impulsive purchase behavior by affecting consumers’ trust and flow experience; Finally, taking consumers who have had live streaming impulsive purchase behavior as the research object, this paper uses structural equation model for empirical analysis, so as to explore the influence mechanism of streamer on consumers’ impulsive purchase behavior in live streaming platform. The innovation of this article mainly lies in dividing the influencing factors of steamers in live streaming into flow experience and turst, considering flow experience and trust as the main influencing mechanisms, and linking consumer impulse purchasing behavior with the influence of streamers.The main purpose of this study is to explore the role of streamers in live streaming, enrich the research scenarios of live streaming, and provide opinions for live streaming platforms and streaming media, promoting the development and construction of the live streaming industry.

## Literature review

### Live streaming

Live shopping is one of the important means of online marketing activities, which has attracted many scholars to conduct research. Unlike traditional online shopping models, live shopping combines live streaming with e-commerce, enabling real-time interaction, display, and promotion of products with users. It has more intuitive and authentic characteristics, and can effectively facilitate the development of online trading activities. Huang Minxue believes that live shopping is carried out online and relies on the role of the streamer to influence user willingness through interaction and other means. It is a combination of social e-commerce and social marketing [8]. Ma based on the U&G theory, explored the halo effect of internet celebrities on consumer purchase intention in live streaming from the perspective of consumer demand, and confirmed that internet celebrities have an important impact on consumer behavior [9]. Gao et al. supported by a fine processing model, believe that information quality in live streaming is influenced by the central path, and streamer characteristics are influenced by the marginal path on consumer purchase and reaction intention [10]. Therefore, in live streaming scenarios, consumer purchasing intentions are influenced by various factors, and understanding the mechanisms of these factors will play a key role in the development of the live streaming industry.

### Source credibility

Source credibility refers to the ability and motivation of the information source to make the information receiver perceive accurate and true information, which has nothing to do with the information itself. Simple clues can affect the source’s credibility, such as the first image, symbols, etc. [11]. High source credibility makes it easier for individuals to be persuaded. On the contrary, low source credibility reduces the probability of individuals being persuaded. In the field of e-commerce, the credibility of information sources is mainly reflected in the audience’s views on the information sources of products and services. Source credibility is often used to study the effectiveness of celebrity endorsement [12]. In the live shopping scene, there are similarities between the roles of the streamer and the celebrity endorser——the streamer plays the role of opinion leader, and its professionalism and authority are the characteristics that consumers pay more attention to [13]. The charisma and professionalism of streamers are important factors that affect consumers’ attitudes and enhance their trust, thereby affecting consumers’ willingness to purchase.

For the live streaming, the relevant information of the streamer has had some impact on consumers before they watch the live streaming because consumers can know the relevant information of the streamer through the platform, such as the corresponding authentication, title, and other personally identifiable information about the streamer given by the official. Consumers can enhance the source credibility of products and services by streamer’s professionalism and personal charm, so as to affect consumers’ purchase intention.

### Social presence theory

Social presence, proposed in 1976, mainly emphasizes the extent to which the media enables users to experience the existence of others [14]. The current sense of social presence has been used in online education, online shopping, human-computer interaction, information systems, and other fields, with rich research results as a reference. It has been mentioned that social presence has a positive impact on consumer purchase intention. In live streaming, social presence also plays an important role. Social presence has a positive impact on the purchase intention of e-commerce live streaming platforms [15], because, in the online environment, social presence can reduce the uncertainty in the online exchange relationship, which has a positive impact on consumer purchase intention [16]. Although offline shopping has more experience advantages than online shopping [17], the emergence of live shopping can largely compensate for the lack of consumer online shopping experience, and the streamer plays an important role in it. In live shopping, the performance of the streamer narrows the distance with consumers to a certain extent and improves consumers’ experience. Through interaction with consumers, it can create a sense of social presence and bring an immersive experience to consumers [18]. Some scholars have proposed that in mobile video live streaming, the interaction of the streamer will have an impact on consumers’ social presence [19]. In addition, the use of effective strategies for interaction can improve the audience’s social sense, thereby effectively affecting their social presence [20]. The emergence of streamers provides a basis for consumers to have a sense of social presence in online shopping. In virtual face-to-face interaction, consumers can experience a strong sense of social presence.

On the one hand, the theory of social presence is used to explain the situation in the live streaming; On the other hand, it serves as the basis for understanding how streamer performance affects consumers’ online impulse purchase behavior.

### Flow experience

“Flow” refers to the feeling that an individual completely invests his energy in a certain activity, whereas “online flow experience” is defined as the state that consumers are completely immersed in a certain online activity [21], which also exists in live shopping. Nowadays, as a trendy online marketing model, live shopping is closely related to the flow experience, and the streamer plays an important role in improving the flow experience of consumers. Previous studies have found that streamer interaction can enhance consumers’ perceived hedonic shopping value [22]. Some scholars have taken shopping enjoyment as a flow experience to explain online unplanned purchases [23]. In addition, streamer interaction can also affect consumers’ emotional and cognitive responses [24]; The entertainment of the streamer can also enable consumers to achieve an excited and happy psychological state in the shopping process [25], to let consumers enter the state of flow. Flow experience is subdivided into two dimensions: enjoyment and focus, which have a positive impact on consumers’ behavior in online stores [26], because these two states are a positive experience for consumers and can trigger positive emotions. Stimulated by positive emotions, consumers will reduce purchase evaluation and make purchase decisions faster, which is easy to cause impulsive purchase desire [27].

Flow experience is an important basis to explain consumers’ live streaming experiences, and it is also an important research direction to explore the impact of consumers’ live impulse purchases.

### Trust

Since the e-commerce platform has become the mainstream development trend of the global retail industry, online shopping has become more and more popular. However, at the same time, due to the virtualization and space-time separation of online shopping, it is easy to cause the asymmetry of product information [28], which affects consumers’ cognition of merchants, improves shopping risk, and reduces the sense of trust. Trust has a direct impact on consumers’ purchase intention in online trading activities [29]. It is also a multidimensional concept, which is divided into cognitive trust and emotional trust [30]. Cognitive trust is a judgment based on the ability and reliability of others, which is an individual’s reasoning based on the behavior information of others in a specific situation [31]; Emotional trust is based on the positive emotions of care and concern shown by the other party [32]. Some scholars have found that cognitive trust and emotional trust have an impact on consumers’ online purchase intention [33].

Therefore, through the disclosure technology of the live streaming platform, consumers can understand the information about the streamer before watching the live streaming, and this kind of information includes the professionalism and personal charisma of the streamer, which will affect consumers’ first image and judgment, change consumers’ cognitive trust and emotional trust in the live streaming scene, and the positive emotions caused by the trust will lead to consumers’ impulsive purchase behavior in the live streaming.

### Impulsive purchase behavior

Impulsive purchase behavior is considered to be unplanned, sudden, and closely related to strong emotions (happiness, excitement) and desires in the shopping process [34]. Impulsive purchase behavior is usually stimulated during shopping [35], which is not only affected by internal factors, including personality, culture, shopping enjoyment tendency, cognition, etc. [36, 37]., but also affected by external factors, such as shopping environment, shopping partner, etc. [38, 39].. In the past, some scholars mentioned that impulsive purchase behavior is affected by many conditions such as economy, place, time, etc., which may lead to the “mixing” of impulsive buying behavior under different buying situations. Therefore, scholars classified impulsive purchase behavior: (A) Pure Impulse Buying: This is truly impulsive buying, the novelty or escape purchase that breaks a normal buying pattern. (B) Reminder impulse buying: their understanding or experience of previous products aroused their memories and stimulated impulse buying. (C) Suggestion impulse buying: this behavior occurs when consumers are unfamiliar with the product they see for the first time and imagine their demand for it. (D) Planned Impulse Buying: Planned impulse buying occurs when the shopper enters the store with some specific purchases in mind, but with the expectation and intention to make other purchases that depend on price specials, coupon offers, and the like [40].

With the continuous development of e-commerce, consumers’ purchase behavior gradually breaks the limitations of time and space, reduces the thinking time of consumers’ purchase decisions, and is more likely to cause impulse purchases behavior [41]. It can be found from the data of “live e-commerce shopping” mentioned above that impulsive purchase behavior accounts for a high proportion of live shopping, which indicates that consumers accept and adapt to the impact of some marketing methods in live shopping [42]. As a new online retail method, live shopping is not only more convenient than offline shopping, but also has a better experience than traditional online shopping, and has the characteristics of high entertainment and interaction. Therefore, the results of previous studies on consumers’ impulsive purchase behavior may not be applicable to live shopping scenarios. Impulse purchasing behavior is common in live streaming, but the cognition of this phenomenon is very scarce. As an indispensable part of live streaming, does the streamer have a corresponding connection with consumers’ impulse purchase behavior?

Paying attention to the impulsive purchase behavior of consumers in the live streaming scenario can not only expand the research on consumer behavior in this scenario but also provide enterprises with an opportunity to cultivate a better consumption experience for consumers, which is also the reason why impulsive purchase behavior is worth studying.

### SOR model

Mehrabian and other scholars put forward the Stimulus-Organism-Response (S-O-R) model. Stimulus refers to the external factors that lead to changes within the individual, Organism refers to the internal psychological state of the individual, and Response refers to the corresponding behavior of the individual stimulated by the stimulation of the external environment. The model connects individual psychology and behavior and explains the mechanism of individual behavior under the stimulation of the external environment. In the field of e-commerce, scholars have taken website features, live shopping features, and scene atmosphere as stimulus variables to study consumers’ corresponding online purchase intention or behavior, including impulsive purchases and continuous purchases. This study uses the S-O-R framework to study the internal state between the streamer and the consumer’s live streaming impulsive purchase behavior, taking the streamer as the stimulation of the external environment, the flow experience and trust as the consumer psychology, and the consumer’s impulsive purchase behavior as the response.

## Hypothetical deduction

From the perspective of the streamer, this study discusses how streamers influence consumers in live streaming. According to the social technology system theory, only by optimizing the social subsystem with emphasis on humanization and the technical subsystem with emphasis on technical capability at the same time can we form a whole [43]. And the streamer not only affects consumers during the live streaming, but also affects consumers before the live streaming. Therefore, we divide the influence of the streamer among consumers into two levels: In the technical aspect of the live streaming platform, the disclosure technology of live streaming enables consumers to pay attention to the streamer information displayed on the platform before live streaming, knowing the characteristics of the streamer, such as the title and personal charm; At the social level, we believe that the performance of the streamer can more effectively change consumers’ experience in live shopping through interaction and entertainment strategies, improving the sense of social presence, and meet consumers’ enjoyment and social needs. Consumers’ trust and flow experience are affected by these two factors respectively, and then affect consumers’ impulsive purchase behavior.

### Streamer characteristics

The streamer’s own characteristics are an important reason to attract consumers to watch live streaming and produce live purchase behavior. This article is divided into two dimensions: the streamer’s charisma and professional characteristics. Some scholars have mined the attributes of e-commerce streamers according to the grounded theory and found that the charm attributes of streamers mainly include appearance, personality, fame, etc., and the “charm” of streamers can change the internal state of consumers, arouse consumers’ sense of excitement, value, and trust, and thus affect consumers’ Online purchase intention [5]. At the same time, the streamer must have professional knowledge about products to better select and recommend [40], attracting the attention of consumers and improving their willingness to watch. The technical availability of the live streaming platform provides more convenience for consumers. With the two characteristics of personal charm and professionalism of the streamer, consumers can understand relevant information before watching the live streaming, to reduce the uncertainty in the process of online purchase.

As mentioned earlier, trust is divided into emotional trust and cognitive trust. Emotional trust includes relational trust and intuitive trust, which involves consumers’ subjective judgment; Cognitive trust includes ability, integrity, and goodwill trust [44]. Through the information presented by the platform, the streamer shows his/her unique identity with personal charm, which will affect the subjective judgment of consumers and cause consumers to intuitively reflect the streamer positively, to improve emotional trust; The perception of the good image of the streamer will enhance the goodwill trust of the streamer, so the streamer’s charm will have an impact on consumers’ emotional trust and cognitive trust; On the other hand, for the professionalism of the streamer, some titles given to the streamer by the platform reflect the professional level and ability of the streamer to a certain extent. Referring to some studies in the online medical community, the indicators of doctors’ ability will be affected by online information [45]. Similarly, in the live streaming environment, such information will affect consumers’ subjective judgment, because no matter whether they understand what these titles represent or not, they will believe that the streamer will act in a trustworthy way to stimulate emotional trust; For the cognitive trust, the professionalism of the streamer can reflect their strength and ability, improving the sense of the reliability of consumers, and promote the cognitive trust of the streamer. Therefore, it is assumed that:

#### H1a

The personality charm of streamers has a positive impact on consumers’ cognitive trust.

#### H1b

The personality charm of streamers has a positive impact on consumers’ emotional trust.

#### H2a

The professional characteristics of streamers have a positive impact on consumers’ cognitive trust.

#### H2b

The professional characteristics of streamers have a positive impact on consumers’ emotional trust.

### Streamer Performance

Consumers’ experience of watching live streaming is not only affected by technical functions, marketing methods, security, and privacy but also the streamer’s performance plays a role to a large extent. In the live streaming environment, the streamer and consumers can interact through the bullet screen. Consumers ask questions on the bullet screen, and the streamer understands consumers’ needs and communicates through the bullet screen. This interaction meets the interactive behavior in the process of information transmission [46], reducing the psychological distance, improving consumers’ sense of presence, and enriching consumers’ experience of online shopping. The interaction between the streamer and consumers will also cause changes in consumers’ emotions and cognition [6].

The live streaming is highly entertaining because the streamer often puts forward interesting activities, such as playing games, lucky draws, etc. For an online shopping environment with entertainment and innovation, this not only has an impact on consumers’ hedonic value but also is related to utilitarian value. The entertainment in this online environment will significantly affect consumers’ perception of information quality [47]. Live streaming can make the atmosphere in the live streaming room relaxed and entertaining through some strategies, whereas a positive network environment can enable individuals to process more information and predict more positive results [48].

In the live streaming situation, the emergence of the interactive form of live streaming provides consumers with a unique experience. In this environment, interaction can enable consumers to enter the state of flow experience [49]. During the live streaming, consumers can enhance their sense of presence, produce a deeper sense of immersion and improve their concentration through frequent communication and interaction with the streamer. In addition, the process of communication, can not only create a more friendlier and cordial environmental atmosphere but also help consumers solve their doubts and problems about products, letting them better enjoy the fun of shopping. Entertainment is not only a feeling beyond experience but also an important core of consumer experience, which has a positive impact on the acquisition of flow experience [50]. The entertainment atmosphere created by the streamer in the live streaming can attract the attention of consumers and improve their concentration in watching the live streaming, and the entertainment experience brought by the streamer can make consumers happy physically and mentally and enjoy it. Therefore, assumptions are put forward:

#### H3a

The interactivity of streamers has a positive impact on consumers’ concentration.

#### H3b

The interactivity of streamers has a positive impact on consumers’ enjoyment.

#### H4a

The entertainment of streamers has a positive impact on consumers’ enjoyment.

#### H4b

The entertainment of streamers has a positive impact on consumers’ concentration.

### Trust and Impulsive Purchase Behavior

In the context of the popularity of the internet, trust is an important factor affecting the whole online purchase process of consumers. High trust can promote the purchase process, whereas low trust will prolong the purchase process. In the e-commerce scene, studies have explored the relationship between trust and purchase behavior: for transactions with uncertainty and information asymmetry in the online environment, consumers’ trust in merchants is the premise [34]. In the process of online shopping, trust has a positive impact on consumers’ purchase intention [51], On the other hand, online impulsive purchase behavior is easily affected by positive emotions caused by consumer experience [52]. The personal charm and professionalism of the streamer will improve the trust in the information source and make consumers feel at ease, thus affecting consumers’ impulse purchase intention. In addition, this trust can affect consumers’ decision-making from other aspects, such as reducing their perception of loss and subjective speculation about the results. Moreover, in the live streaming situation, consumer trust will play an important role in promoting the rapid conclusion of the transaction, which is more conducive to the generation of an impulsive purchase. Therefore, the more consumers trust the streamer, the more impulsive purchase behavior will be aroused in the process of watching the live streaming. Therefore, it is assumed that:

#### H5a

cognitive trust caused by streamer features has a positive impact on consumers’ impulsive purchase behavior.

#### H5b

emotional trust caused by streamer features has a positive impact on consumers’ impulsive purchase behavior.

### Flow Experience and Impulsive Purchase Behavior

The flow experience obtained by consumers in the live streaming has a positive impact on consumers’ impulsive purchase behavior. Some studies have confirmed that there is a significant correlation between emotion and consumers’ impulsive purchases [53]. Positive emotion positively affects consumers’ impulsive purchase behavior, and flow experience can improve consumers’ positive emotions. In the state of flow, people can enjoy the peak of the experience, making their hearts full and harmonious, focused, and pleasant. This experience full of positive emotions affects consumers’ impulsive purchase behavior. The performance of the streamer in live streaming attracts the attention of consumers. On the one hand, consumers pay attention to the relevant information about products, on the other hand, the live streaming content can also bring fun to consumers. Therefore, consumers are satisfied with utilitarianism and pleasure, which is more likely to cause impulse purchase desire. In the internet environment, the purchase is not limited by space and time, so the impulse purchase desire caused is more likely to change into impulse purchase behavior. To sum up, it is proposed that streamer performance is easier to bring a good experience, resulting in consumers’ impulsive purchase behavior. Therefore, it is assumed that:

#### H6a

the enjoyment caused by the streamer’s performance has a positive impact on consumers’ impulsive purchase behavior.

#### H6b

the concentration caused by the streamer’s performance has a positive impact on consumers’ impulsive purchase behavior.

**Fig. 1:**
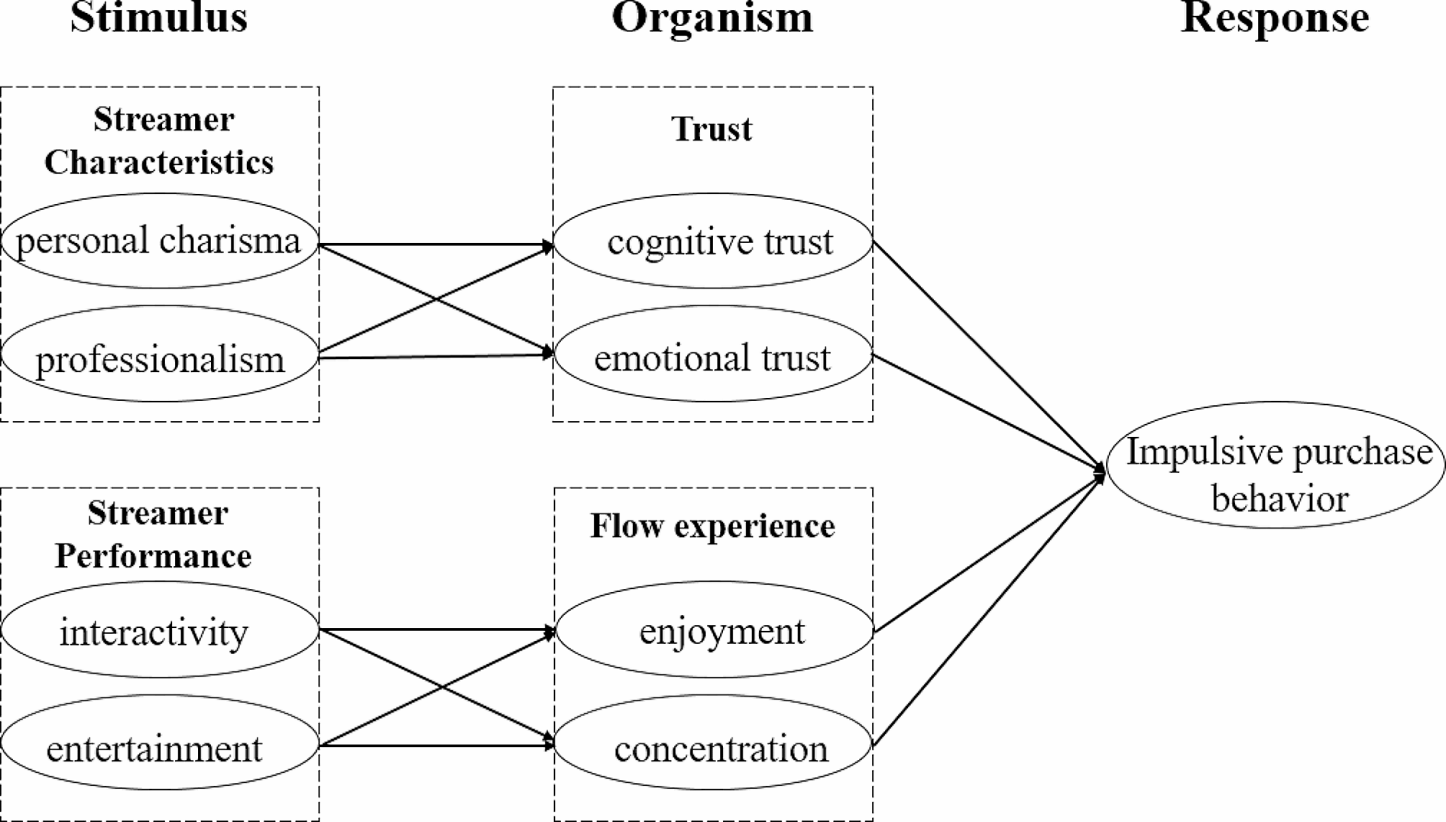
Research model

Figure 1 presents the basic framework.

## Research design

### Measures

The questionnaire is mainly divided into two parts: the first part is mainly the measurement of variables. Based on using the maturity scale, the items are adjusted and modified appropriately. For the foreign language scale in the literature, multiple translations and back translations are carried out to ensure the accuracy of language expression. As shown in Table 1.

**Table 1:** Measurement scales

Constructs	Items
**Personal Charisma**[22, 54]	
PC1	The appearance of the streamer attracts me
PC2	The streamer is very charming
PC3	Enjoy the streamer
PC4	The streamer can attract my attention more
**Professionalism** [43, 55]	
PF1	When I see the official authentication of the streamer’s identity, I think the streamer has a wealth of knowledge in the field of this product
PF2	When I see the streamer’s self-introduction, I think the streamer is familiar with the products he recommends
PF3	When I see that the streamer has a specific domain logo(like Beauty Blogger), I think the streamer is competent in the product field
**Interactivity** [56, 57]	
IT1	The streamer has good interaction with me
IT2	The streamer can respond and answer the barrage in time
IT3	The streamer can respond and answer the bullet screen in time
IT4	The distance between me and the streamer is shortening
**Entertainment** [25, 57]	
EN1	I think the streamer’s performance is very interesting
EN2	I think the streamer’s performance makes me relax
EN3	I think the streamer’s performance makes me happy
**Emotional Trust** [58, 59, 60]	
ET1	The streamer will treat me with enthusiasm and care
ET2	The streamer will kindly reply to my questions on the bullet screen
ET3	I will freely share my thoughts and feelings with the streamer
ET4	If I can’t watch the live streaming of the streamer again, I will feel sad
**Cognitive Trust** [58, 59, 60]	
CT1	I will rely on the streamer to provide information about the product for analysis and judgment before buying the product
CT2	I have sufficient reasons to believe in the professional knowledge and ability of the streamer
CT3	I will buy products according to the information provided by the streamer
CT4	The streamer treats our interactions with professionalism and dedication
**Enjoyment** [27, 61]	
EJ1	Watching live streaming is my favorite activity
EJ2	It’s interesting for me to watch live streaming
EJ3	It’s very attractive for me to watch live streaming
EJ4	It makes me feel happy to watch the live streaming
EJ5	It’s exciting to watch live streaming
**Concentration** [27, 61]	
CC1	My attention is focused on the live streaming
CC2	I won’t think about anything else when watching the live streaming
CC3	I can hardly be distracted when watching the live streaming
CC4	I’m absorbed in the live streaming
**Impulsive Purchase Behavior** [61, 62]	
IP1	I often buy things I didn’t intend to buy in the live streaming
IP2	In the live streaming, I often find some products that I don’t plan to buy
IP3	I often buy a product without thinking in the live streaming

### Personal charisma

The streamer’s personal charm mainly refers to the research scale of Hyun Jung Park and Fengjun Liu [22, 54],which comprises four items (e.g.,“The appearance of the streamer attracts me”).Participants rated each item on a 5-point Likert scale, ranging from 1 (very disagree) to 5 (very agree).The internal consistency coefficient of this questionnaire in our study was 0.817.

### Professionalism

Professionalism mainly refers to the research scale of Yang Nan and other scholars [43, 55],which comprises three items (e.g.,“When I see the official authentication of the streamer’s identity, I think the streamer has a wealth of knowledge in the field of this product”).Participants rated each item on a 5-point Likert scale, ranging from 1 (very disagree) to 5 (very agree).The internal consistency coefficient of this questionnaire in our study was 0.76.

### Interactivity and entertainment

Interactivity and entertainment mainly refer to the research results of Chia Chen Chen, Liu Zhongyu and other scholars [56, 57],which comprises four items (e.g.,“The streamer has good interaction with me”)and three items(e.g.,“I think the streamer’s performance is very interesting”)Participants rated each item on a 5-point Likert scale, ranging from 1 (very disagree) to 5 (very agree).These two internal consistency coefficient of this questionnaire in our study were 0.75 and 0.788.

### Emotional Trust and Cognitive Trust

Emotional Trust and Cognitive Trust mainly refers to the research scale of Nick hajli, apiradee wongkitrungrueng, Devon Johnson and other scholars [58–60],which comprises four items (e.g.,“The streamer will treat me with enthusiasm and care”)and four items(e.g.,“I will rely on the streamer to provide information about the product for analysis and judgment before buying the product”)Participants rated each item on a 5-point Likert scale, ranging from 1 (very disagree) to 5 (very agree).These two internal consistency coefficient of this questionnaire in our study were 0.781 and 0.746.

### Enjoyment and concentration

Enjoyment and Concentration mainly refers to the research scale of Ettis and other scholars [27, 61],which comprises five items (e.g.,“Watching live streaming is my favorite activity”)and four items(e.g.,“My attention is focused on the live streaming”).Participants rated each item on a 5-point Likert scale, ranging from 1 (very disagree) to 5 (very agree).These two internal consistency coefficient of this questionnaire in our study were 0.839 and 0.895.

### Impulsive purchase behavior

Impulsive purchase behavior refers to the research results of Wu and other scholars [27, 61],which comprises five items (e.g.,“I often buy things I didn’t intend to buy in the live streaming”).Participants rated each item on a 5-point Likert scale, ranging from 1 (very disagree) to 5 (very agree).The internal consistency coefficient of this questionnaire in our study was 0.749.

The second part is the collection of personal basic data, such as gender, income, etc. The questionnaire adopts the Likert 5 scale to measure the above items, “1” means “very disagree” and “5” means very agree. After sorting out the first draft of the questionnaire, a simple pre-test was carried out on the questionnaire: recruit in-depth users of live shopping to evaluate the questionnaire, take the purchase frequency and purchase times in the live streaming as the basis to judge the in-depth users, and evaluate whether the situation of the questionnaire is suitable or omitted, whether the items are clear and unambiguous, and whether the description of the questionnaire is close to the real situation, Finally, adjust the questionnaire to make it easy to understand.

### Procedure

The present study was conducted in accordance with the recommendations of the Ethics Committee of the School of Psychology, Sichuan Agricultural University, and approved by the same committee.And the study was performed in line with the principles of the Declaration of Helsinki.

After revising the questionnaire, a total of 375 electronic questionnaires were distributed through the “Questionnaire Star” platform on Weibo, QQ groups, and live streaming communities. After removing the samples that did not produce impulsive purchase behavior in the “live shopping” and some invalid samples, 250 valid questionnaires were finally recovered. The respondents were people who had impulsive purchase behavior on the live platform, there are corresponding screening questions in the questionnaire (have you ever had impulsive purchase behavior in “live shopping?) And attention-check questions to ensure the quality of the questionnaire.

From the 250 valid data collected(see Table 2), the number of women is too large, accounting for the majority (78.8%), on the contrary, the number of men is relatively low (21.2%), but previous studies have found that women are often more likely to buy impulsively than men [53], so it is reasonable. In terms of age, most people are concentrated in the range of 18–35 years old. According to the results of the “online survey report on consumer satisfaction of live e-commerce shopping”, post-80s, the 90s, and 00s are the main force of online shopping at present, so the age structure should also be appropriate. In general, in this survey, the collection of samples is roughly consistent with the corresponding literature and data. The following are the descriptive statistics of the samples:

**Table 2:** Sample characteristics

Characteristics	Category	Frequency	Percentage
Gender	Male	53	21.2%
	Female	197	78.8%
Age	Less than 18 years	6	2.4%
	18–25 years	204	81.6%
	26–35 years	30	12%
	36–45 years	7	2.8%
	Over 46 year	3	1.2%
Consumption levels	Below 1000 Yuan	21	8.4%
	1000–3000 Yuan	195	78%
	3001–5000 Yuan	27	10.8%
	5001–8000 Yuan	4	1.6%
	Over 8000	3	1.2%

## Analysis of empirical results

### Reliability and effectiveness

In terms of reliability and validity, exploratory factor analysis and confirmatory factor analysis were carried out by spss22.0 and amos22.0 (see Table 3). In terms of reliability, taking the Cronbach index as the test index, it is found that the α value of all variables is higher than 0.7(above 0.7 is acceptable), indicating that the reliability of the item meets the requirements, and the KMO value in the validity analysis is 0.867, indicating that it is suitable for factor analysis, and the Standardized loading of each dimension is also greater than the threshold value of 0.5(above 0.5 is acceptable); In addition, the values of composite reliability C.R are higher than the threshold of 0.7(above 0.7 is acceptable), which indicates that there is no problem with the internal consistency of variables in the questionnaire.

**Table 3:** Standardized load and reliability

Variable	Standardized Loadings	α	C.R	AVE
Personal Charisma	0.617	0.817	0.7820	0.5477
	0.794			
	0.795			
	0.719			
Professionalism	0.720	0.760	0.7612	0.5153
	0.696			
	0.737			
Interactivity	0.699	0.750	0.7519	0.5033
	0.665			
	0.761			
Entertainment	0.714	0.788	0.7975	0.5684
	0.731			
	0.813			
Cognitive Trust	0.712	0.746	0.7523	0.5034
	0.675			
	0.740			
Emotional Trust	0.653	0.781	0.7872	0.5540
	0.789			
	0.783			
Enjoyment	0.757	0.839	0.8397	0.5672
	0.735			
	0.789			
	0.730			
Concentration	0.683	0.895	0.8977	0.6893
	0.833			
	0.906			
	0.881			
Impulsive Purchase Behavior	0.799	0.749	0.7605	0.5161
	0.648			
	0.700			

As for the convergent validity, it can be found from Table 3 that the AVE of each variable is greater than 0.5(above 0.5 is acceptable), which effectively ensures the structural validity. For discriminant validity, the diagonal of Table 4 is the square root of AVE, and the square root of AVE of each dimension is greater than the correlation coefficient between dimensions. Therefore, each dimension of this study has sufficient discrimination from other dimensions, indicating good discriminant validity.

**Table 4:** Correlation analysis

	P.C	PRO	INT	ENT	C.T	E.T	ENJ	CON	IMP
P.C	0.740								
PRO	0.372	**0.717**							
INT	0.338	0.276	**0.709**						
ENT	0.421	0.454	0.482	**0.753**					
C.T	0.377	0.402	0.407	0.463	**0.709**				
E.T	0.342	0.232	0.508	0.428	0.363	**0.744**			
ENJ	0.367	0.326	0.395	0.493	0.343	0.495	**0.753**		
CON	0.310	0.230	0.390	0.314	0.449	0.434	0.544	0.83	
IMP	0.259	0.201	0.309	0.223	0.346	0.241	0.264	0.398	0.718

The structural equation model was evaluated by confirmatory factor analysis with amos22.0. The results(see Table 4) showed that x^2^ / DF value was 2.015, and other fitting indexes (CFI = 0.883; IFI = 0.885; GFI = 0.829; RMSEA = 0.064) were in line with relevant indexes. Therefore, the model showed acceptable goodness of fit.

In addition, this study discusses the multicollinearity problem in the study by calculating the Variance Inflation Factor (VIF) [63]. The results show that the VIF values of all variables are less than 3, which indicates that there is no multicollinearity problem in this study.

### Common method deviation

Since this study is to collect data through questionnaires, which are filled in by the same participants, there may be potential problems of common method deviation. Two methods are used to verify respectively. One is Harman’s single factor test; the Second, it is verified by a one-dimensional model test in CFA [64]. In Harman’s single factor validation, the largest extraction factor explained 28.2% of the total variance, which was 40% lower than the threshold; In CFA verification, the fitting index of one-dimensional model (x^2^ / DF = 4.521, RMSEA = 0.119) is worse than that of measurement model (x^2^ / DF = 1.638, RMSEA = 0.051). In conclusion, the proposed common method deviation will not affect the results of this study.

### Structural model analysis

**Fig. 2:**
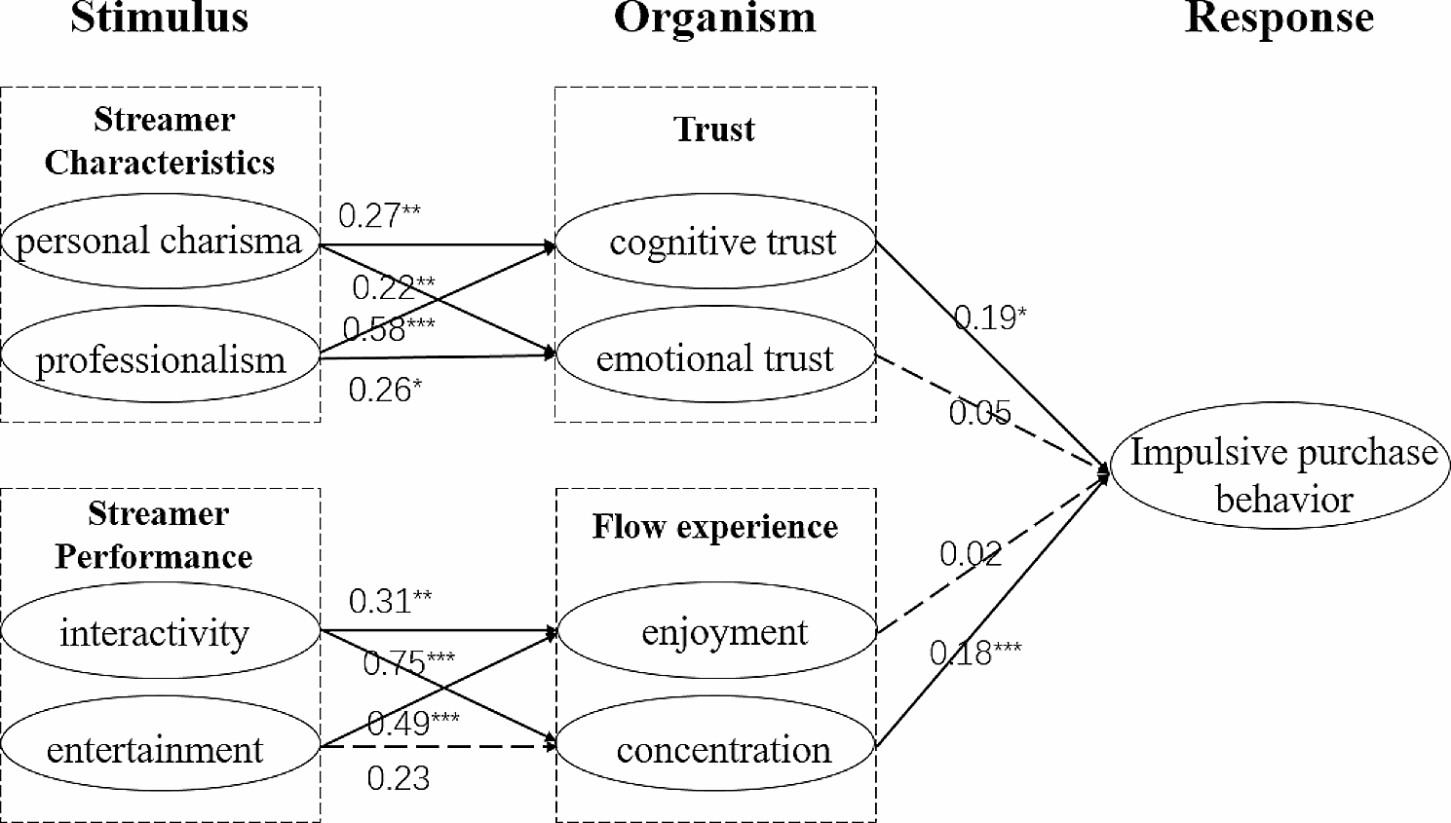
Model analysis results

In the aspect of model path analysis, the amos22.0 tool is used to verify the proposed assumptions. Firstly, for the personal charm and professionalism in the streamer characteristics, the streamer’s personal charm has great influence on cognitive trust (β = 0.27^**^, *P* < 0.01) and emotional trust (β = 0.22^**^, *P* < 0.01); Similarly, professionalism is both about cognitive trust( β = 0.58^***^, *P* < 0.001) or emotional trust (β = 0.26^*^, *P* < 0.05). Secondly, in the streamer performance, interactivity can effectively affect consumers’ flow experience and enjoyment (β = 0.31^**^, *P* < 0.01) and concentration (β = 0.75^***^, *P* < 0.001), whereas entertainment only affects consumers’ enjoyment (β = 0.49^***^, *P* < 0.001), but the effect on the concentration is not significant. Finally, compared with emotional trust, cognitive trust (β = 0.19^*^, *P* < 0.05) effectively affects consumers’ impulsive purchase behavior, whereas, in the flow experience, the enjoyment can affect consumers’ impulsive purchase behavior more than the concentration (β = 0.18^***^, *p*<0.001). Table 5; Fig. 2 shows the final path analysis results.

**Table 5:** Path analysis

Hypothesis	β	T	Result
H1a	0.27^**^	3.164	supported
H1b	0.22^**^	2.932	supported
H2a	0.58^***^	3.956	supported
H2b	0.26^*^	2.269	supported
H3a	0.31^**^	2.681	supported
H3b	0.75^***^	3.950	supported
H4a	0.49^***^	4.256	supported
H4b	0.23	1.260	Not supported
H5a	0.19^*^	2.323	supported
H5b	0.05	0.466	Not supported
H6a	0.02	0.241	Not supported
H6b	0.18^***^	3.527	supported

*p<0.05, **p<0.01, ***p<0.001

**Table 6:** Mediating effect test of cognitive trust (P.C)

	I.P.B		C.T		I.P.B	
	β	t	β	t	β	t
P.C	0.2671	4.2208^***^	0.3518	6.4186^***^	0.1678	2.5279^*^
C.T					0.2821	3.9602^***^
R^**2**^	0.067		0.1425		0.1227	
F	17.8151		41.199		17.2767	

### The mediation tests

This study uses model 4 [65] of the process plug-in in spss22.0 to test the mediating effect in the relationship between personal charm, professionalism, interaction, and impulsive purchase behavior. All test results are as follows:

As shown in Table 6, the influence of personal charm on impulsive purchase behavior is significant(β = 0.2671, t = 4.2208, *P* < 0.001). When the mediating variable cognitive trust is added, the influence of personal charm on impulsive purchase behavior is also significant (β = 0.1678, t = 2.5279, *P*<0.05). The influence of personal charm on cognitive trust is significant (β = 0.3518, t = 6.4186, *P* < 0.001), and cognitive trust is also significant for impulsive purchase behavior (β = 0.2821, t = 3.9602, *p*<0.001).

In addition, Table 7 shows that the direct effect of personal charm on impulsive purchase behavior and the mediating effect of cognitive trust does not contain 0 in the Lower and Upper limits of 95% confidence interval of bootstrap (0.152$\sim$0.3524, 0.0354$\sim$0.1696), which shows that personal charm can not only directly affect impulsive purchase behavior, but also affect the impulsive purchase behavior through the mediating effect of cognitive trust.

**Table 7:** Effect analysis

	Effect	BootSE	BootLLCI	BootULCI	
Total effect	0.2671	0.087	0.105	0.447	
Direct effect	0.1678	0.856	0.152	0.3524	63%
Mediation	0.0992	0.0344	0.0354	0.1696	37%

#### NOTE1

I.P.B = Impulsive Purchase Behavior, C.T = Cognitive Trust, P.C = Personal Charisma, P.F = Professionalism, C.C = Concentration, I.T = Interactivity.

#### NOTE2

p^*^<0.05, p^**^<0.01, p^***^<0.001.

According to the results in Table 8, professionalism has a significant impact on impulsive purchase behavior (β = 0.2662, t = 3.2371, *P* < 0.01). After adding the mediating variable cognitive trust, professionalism has no significant effect on impulsive purchase behavior (β = 0.1172, t = 1.35, *P* > 0.05), while cognitive trust has a significant impact on impulsive purchase behavior (β = 0.3106, t = 4.2743, *P* < 0.001), and the influence of professionalism on cognitive trust is significant(β = 0.4796, t = 6.9039, *p*<0.001).

**Table 8:** Mediating effect test of cognitive trust (P.F)

	I.P.B		C.T		I.P.B	
	β	t	β	t	β	t
P.F	0.2662	3.2371^**^	0.4796	6.9039^***^	0.1172	1.35
C.T					0.3106	4.2743***
R^2^	0.0405		0.1612		0.1066	
F	10.4789		47.6642		14.739	

Therefore, according to Table 9, the mediating effect in the influence of professionalism on impulsive purchase behavior does not contain 0 in the Lower and Upper limits of 95% confidence interval of bootstrap, indicating that the mediating effect exists. However, the direct effect includes 0 (-0.717 $\sim$ 0.2816) in the Lower and Upper limits of 95% confidence interval of bootstrap. Strictly speaking, cognitive trust plays a full-mediation role in the influence of professionalism on impulsive purchase behavior.

**Table 9:** Effect analysis

	Effect	BootSE	BootLLCI	BootULCI	
Total effect	0.2662	0.091	1.841	3.225	
Direct effect	0.1172	0.91	-0.717	0.2816	44%
Mediation	0.149	0.52	0.619	0.2651	56%

It can be found from Table 10 that interactivity has a significant impact on impulsive purchase behavior (β = 0.3586, t = 5.1143, *P* < 0.001). After adding mediating variable concentration, the influence of interactivity on impulsive purchase behavior is also significant(β = 0.2102, t = 2.9053, *p*<0.01). The influence of interactivity on concentration is also significant(β = 0.5681, t = 6.6746, *p*<0.001); The effect of concentration on impulsive purchase behavior is also significant(β = 0.2611, t = 5.2518, *p*<0.001).

**Table 10:** Mediating effect test of concentration

	I.P.B		C.C		I.P,B	
	β	t	β	t	β	t
I.T	0.3586	5.1143^***^	0.5681	6.6746^***^	0.2102	2.9053^**^
C.C					0.2611	5.2518^***^
R^2^	0.0954		0.1523		0.1863	
F	26.1558		44.5503		28.2701	

According to Table 11, it can be found that the direct effect of interaction on impulsive purchase behavior and the mediating effect of concentration dose not include 0 in the Lower and Upper limits of 95% confidence interval of bootstrap, indicating that interaction can not only directly affect impulsive purchase behavior, but also affect the impulsive purchase behavior through the mediating effect of concentration.

**Table 11:** Effect analysis

	Effect	BootSE	BootLLCI	BootULCI	
Total effect	0.3586	0.078	0.205	0.513	
Direct effect	0.2102	0.0871	0.0363	0.3844	59%
Mediation	0.1483	0.441	0.708	0.2424	41%

## Conclusion

As the current hot spot, live streaming is not only a new marketing method of online shopping but also a new trend in the retail industry in the future. As a key factor in live streaming, the streamer plays an important role. Based on the SOR theoretical framework, this paper integrates the source credibility theory, flow experience theory, and social presence theory, focuses on the characteristics and performance of the streamer before and during the live streaming, and finally discusses the relationship between the streamer and consumers’ impulsive purchase behavior.

### Research findings

This study investigates how the streamer’s personal charisma, professionalism, interactivity, and entertainment can improve consumers’ trust and flow experience, so as to promote consumers’ impulsive purchase behavior. The empirical results support most of the assumptions in this study. First of all, the personal charisma and professionalism of the streamer have a significant effect on improving consumer trust: because the appearance, reputation, and other factors of the streamer will affect the first image of consumers, which is easy to make consumers trust the streamer more intuitively; With the help of live streaming disclosure technology, the platform can fully display the relevant professional information of the streamer, such as the special signs of the professional field. Such information shows the ability of the streamer to a certain extent, and the professional information of these streamers also provides the basis for consumer trust.

Secondly, compared with emotional trust, cognitive trust has a more significant impact on impulsive purchase behavior. Today, online consumers have become more rational. The factors they consider when buying products may pay more attention to the product itself, weaken the influence outside the product, and judge whether they need to buy more based on objective conditions. This also shows that the quality and concept of online consumption are constantly improving, and consumers are more inclined to their own personalized needs when buying products.

In addition, the interactivity and entertainment of streamers have a certain relationship with the flow experience of consumers. The interactivity of the streamer in live streaming has a significant impact on consumers’ enjoyment and concentration. Because the interaction, communication, and corresponding activities in the live streaming can provide a good atmosphere for the live streaming room, which can not only improve the attention of consumers but also activate consumers’ mood to watch the live streaming. However, the entertainment shown by the streamer has no significant impact on consumers’ concentration, only on the enjoyment. This study believes that the main purpose of watching e-commerce live streaming is to buy needed products. However, although the entertainment of live streaming can effectively improve consumers’ experience, excessive entertainment live streaming may deviate from consumers’ initial shopping needs, thereby reducing consumers’ attention to live streaming.

Finally, in the concentration and enjoyment caused by streamer performance, only the concentration has a significant impact on impulsive purchase behavior. Consumers’ positive emotions in live streaming can effectively promote their impulsive purchase behavior. However, in the live streaming situation, the positive emotions caused by the enjoyment brought by the streamer to consumers may not be enough to arouse consumers’ impulsive purchase desire, and the concentration makes consumers more immersed in the live streaming, which is more likely to cause impulsive purchase behavior.

### Theoretical contributions and practical contributions

Although previous studies have discussed the relationship between streamers and consumer behavior, few articles have talked about the impact of streamers on consumer impulsive purchase behavior, and the traditional research on consumer impulsive purchase behavior is not fully applicable to live streaming scenarios, and the results of this paper will have corresponding significance for the development of academia and live streaming.

The theoretical contributions are that: First of all, this study enriches the existing research in the field of the live streaming, combs the relationship between the influence of streamers and consumers’ impulsive purchase behavior, and constructs a new model based on S-O-R model, using source credibility theory, flow experience theory, and social presence theory to explore the influence mechanism of streamers on consumers’ impulsive purchase behavior. This paper evaluates the important role of streamers in consumer behavior with their personal charisma, professionalism, interactivity, and entertainment, and provides a new perspective.

Secondly, this study divides the influence factors of the streamer into two dimensions, namely, the influence of the streamer before the live streaming (streamer characteristics) and the influence of the streamer in the live streaming (streamer performance), which strengthens the understanding of the influence level of the streamer. This consideration is mainly based on “the influence of the streamer does not only exist in the live streaming, but also the influence of the user on the information disclosure of the streamer on the live streaming platform”, Such dimensional classification can provide new ideas for the future streamer research;

In addition, the research results of this paper show that emotional trust and enjoyment have no positive impact on consumers’ impulsive purchase behavior, but this is contrary to the previous research results on online impulsive purchase behavior. On the one hand, it shows that there are differences between the influencing factors of impulsive purchase behavior in live streaming scenes and traditional online impulsive purchase behavior. On the other hand, it also shows that consumers are more likely to be affected by cognitive trust and concentration in the trust and flow experience in the live streaming, so this study further enriches the application of trust and flow experience in the live streaming scene, and proposes that cognitive trust and concentration can more cause consumers’ impulsive purchase behavior in the live streaming; Finally, cognitive trust plays a complete mediation role in the influence path of professionalism on impulsive purchase behavior, proving the mechanism between professionalism and impulsive purchase behavior, indicating that the professionalism of streamer affects impulsive purchase behavior by improving consumers’ cognitive trust. This discovery adds new knowledge to the influence of streamers in the live shopping environment.

The practical contributions are that: For the live streaming platform, the platform should have corresponding thresholds when selecting the streamer, such as the streamer’s image, professional ability, communication and interaction, and the planning ability of live streaming marketing activities. In addition, it should also carry out some professional training for the streamer, such as strengthening the understanding of products, improving their own image, improving communication skills, increasing communication activities, etc., because, from the research results, streamer characteristics and performance are effective factors that affect consumers, and streamers are the key media in live streaming, which not only plays an important role in attracting consumers but also plays a key role in promoting consumers’ purchase behavior. In terms of some system functions of the platform, this study suggests that the platform should do a good job in the publicity of streamers, improving the information disclosure function of the platform, letting more consumers understand and discover the relevant information of streamers, and provide consumers with a reference to watch live streaming to a certain extent, such as the domain classification, identity introduction, evaluation and other information of streamers.

For streamers, only by improving their comprehensive ability can they stand out in the competition of live streaming. On the one hand, streamers should strive to improve themselves, show their beautiful image to the audience, strengthen professional training, improve professional ability, improve consumers’ trust in streamers, and pay attention to the interaction with consumers in live streaming; On the other hand, the streamer should make effective use of the platform, show themselves with the help of the platform, highlight their own specificity and ability, attract consumers, improve consumer trust, and then bring a better experience to consumers through interactive ways such as bullet screen, so that consumers can immerse themselves in the live streaming and retain the consumers in the live streaming.

### Limitations and Future Research

Based on the live shopping environment, this paper discusses the relationship between the streamer and consumers’ impulsive purchase behavior, but there are still some limitations in the research. First of all, this study was sampled in China and did not take into account the cultural background of different countries and the actual development of live streaming e-commerce. Therefore, the selection of survey samples still lacks universality. Future research can consider collecting the views of consumers in many regions on the relationship between live streaming streamers and impulsive purchase behavior, to further improve the research.

Secondly, impulse purchase behavior in live streaming is not only affected by flow experience and trust but also affected by many other factors, such as satisfaction and time pressure. In the future, we can try more variables from different scenes to further discuss the impact of streamers on consumers’ impulse purchase behavior in live streaming; Besides, although the two stimuli of streamer characteristics and streamer performance are considered, the impact caused by the relationship between live streaming products and streamer is not considered. Therefore, the impact of the relationship between streamers and products on consumers can be considered in the future.

In addition, e-commerce platforms (Taobao, jd.com, etc.) and social media (Tiktok, Kwai, etc.) do not have a classification for live streaming. For two different tape platforms, consumers’ needs and perceptions are different. Therefore, the key points that influence the behavior of consumers are different. In the future, we can study the key factors affecting consumer behavior in different types of platforms.

Finally, impulse-induced purchase will not be a long-term development for both the streamer and the platform. It only has miraculous effects in specific scenes (such as unsalable agricultural products and promotional activities). Future research can consider relevant behaviors such as continuous purchase and repurchase, so as to promote the long-term development of the live streaming platform.

### Electronic supplementary material

Below is the link to the electronic supplementary material.

Supplementary Material 1

## Data Availability

All data analysed during this study are included in this published article.
