# Supplementary material for: Why consumers have impulsive purchase behavior in live streaming: the role of the streamer
Source: BMC Psychol. 2024 Mar 6;12:129. doi: 10.1186/s40359-024-01632-w (PMC10918902; doi:10.1186/s40359-024-01632-w)
Supplement: Supplementary file 1 — Supplementary Material 1 [file 40359_2024_1632_MOESM1_ESM.docx]

**Research Scales**

**A. Personal Charisma Scales**

1. The appearance of the streamer attracts me.

2. The streamer is very charming.

3. Enjoy the streamer.

4. The streamer can attract my attention more.

**B. Professionalism scale**

1. When I see the official authentication of the streamer's identity, I think the streamer has a wealth of knowledge in the field of this product.

2. When I see the streamer's self-introduction, I think the streamer is familiar with the products he recommends.

3. When I see that the streamer has a specific domain logo(like Beauty Blogger), I think the streamer is competent in the product field.

**C. Interactivity scale**

1. The streamer has good interaction with me.

2. The streamer can respond and answer the barrage in time.

3. The streamer can respond and answer the bullet screen in time.

4. The distance between me and the streamer is shortening.

**D. Entertainment scale**

1. I think the streamer's performance is very interesting.

2. I think the streamer's performance makes me relax.

3.I think the streamer's performance makes me happy.

**E. Emotional Trust scale**

1. The streamer will treat me with enthusiasm and care.

2. The streamer will kindly reply to my questions on the bullet screen.

3. I will freely share my thoughts and feelings with the streamer.

4. If I can't watch the live streaming of the streamer again, I will feel sad.

**F. Cognitive Trust scale**

1. I will rely on the streamer to provide information about the product for analysis and judgment before buying the product.

2. I have sufficient reasons to believe in the professional knowledge and ability of the streamer.

3. I will buy products according to the information provided by the streamer.

4. The streamer treats our interactions with professionalism and dedication.

**G. Enjoyment scale**

1. Watching live streaming is my favorite activity.

2. It's interesting for me to watch live streaming.

3. It's very attractive for me to watch live streaming.

4. It makes me feel happy to watch the live streaming.

5. It's exciting to watch live streaming

**F. Concentration scale**

1. My attention is focused on the live streaming.

2. I won't think about anything else when watching the live streaming.

3. I can hardly be distracted when watching the live streaming.

4. I'm absorbed in the live streaming.

**G. Impulsive Purchase Behavior scale**

1. I often buy things I didn't intend to buy in the live streaming.

2. In the live streaming, I often find some products that I don't plan to buy.

3. I often buy a product without thinking in the live streaming.
